# Decoding the Flavor Code of Fresh and Dried *Tengjiao* (*Zanthoxylum armatum* DC.) for Preparing Fried *Tengjiao* Oil Through Molecular Sensory Science

**DOI:** 10.3390/foods15081326

**Published:** 2026-04-10

**Authors:** Tianyu Dong, Panpan Wu, Jie Sun, Haitao Chen, Shuqi Wang

**Affiliations:** Beijing Key Laboratory of Flavor Chemistry, Beijing Technology and Business University, Beijing 100048, China; dty19991030@163.com (T.D.); 18617240636@163.com (P.W.); wangshuqi@btbu.edu.cn (S.W.)

**Keywords:** fried *Tengjiao* (*Zanthoxylum armatum* DC.) oil, molecular sensory science, key flavor compounds, partial least squares regression (PLSR), enantiomer

## Abstract

Fried *Tengjiao* oil is commonly used for seasoning spicy dishes, and both fresh and dried *Tengjiao* are used in its preparation. However, the flavor differences between fried *Tengjiao* oils prepared from these two types of raw materials have not yet been studied. The aim of this study was to compare and analyze the flavor differences between fresh fried *Tengjiao* oil (FFTO) and dried fried *Tengjiao* oil (DFTO). In this study, molecular sensory science was employed to reveal the flavor differences between the two at the molecular level. FFTO had a stronger pepper and spice aroma, while DFTO exhibited a more marked oily aroma. A total of 82 volatile compounds were identified via SAFE-GC-MS (solvent-assisted flavor evaporation–gas chromatography–mass spectrometry). Based on AEDA (aroma extract concentration analysis), 36 aroma-active compounds with FD ≥ 27 were accurately quantified. Following the AEDA, OAV analysis, and recombination experiments and omission tests, linalool and β-caryophyllene were identified as key flavor compounds in FTOs. α-thujone, 3-buten-1-yl isothiocyanate, citronellal, linalyl acetate, and 3-phenylpropionitrile were key flavor compounds in FFTO, and *β*-pinene, *α*-terpinene, *β*-phellandrene, and 3-ethyl-2,5-dimethylpyrazine were key flavor compounds in DFTO. Finally, chiral analysis suggests that the ratio of linalool enantiomers may be the potential cause of the flavor differences between FFTO and DFTO. This study provides theoretical guidance for the industrial production of FTO.

## 1. Introduction

*Zanthoxylum armatum* DC. (ZA), also known as *Tengjiao*, is a small tree or shrub belonging to the genus Zanthoxylum in the Rutaceae family [1]. ZA is originally from China, primarily distributed in southwestern regions such as Sichuan, Chongqing, and Guizhou [2]. In addition, it also has a certain range of distribution in Japan, Republic of Korea, Nepal, India, and Pakistan [3]. It is one of the most widely used seasonings and also possesses certain medicinal value, being used to treat conditions such as colds, toothaches, fevers, indigestion, and cholera [4]. When it comes to Chinese ZA, Sichuan ZA is particularly famous for its special flavor and quality. Specifically, Meishan City in Sichuan Province is situated at the junction of the Sichuan Basin and the Western Sichuan Plateau. Its climate is mild, with pleasant weather year-round, abundant rainfall, and ample sunshine, making it particularly suitable for the growth of Sichuan peppers. Hongya County, under the jurisdiction of Meishan City, has a high forest coverage rate and is well known as a “natural oxygen bar” and the “hometown of *Tengjiao*”. As a result, Hongya *Tengjiao* is widely used in Sichuan cuisine and processed to make products such as *Tengjiao* oil and *Tengjiao* sauce.

In Chinese cuisine, both fresh and dried ZA are commonly used as ingredients in cooking, endowing dishes with a fresh, green aroma and a numbing, spicy taste. With the innovative development of the restaurant industry, *Tengjiao* has found increasingly diverse applications in culinary products. For instance, fried *Tengjiao* oil (FTO) can be used to season rice noodles, hot pot, and other dishes. Additionally, the special green pepper roasted fish is particularly popular among young consumers. It is noteworthy that both dried and fresh *Tengjiao* are utilized in these cooking processes. Therefore, the scientific basis for using both of them simultaneously in the cooking process is an area worth exploring.

Currently, research on ZA has primarily centered on its biological activity, volatile flavor compounds, fagaramide (the numbing component), and extraction processes. Previous studies have demonstrated that ZA exhibits anti-inflammatory, antioxidant, antispasmodic, antibacterial, antiprotozoal, antiviral, anthelmintic, antinociceptive, antiproliferative, antidiabetic, antidepressant, antipyretic, cytotoxicity, hepatoprotective, insecticidal, larvicidal, phytotoxicity, piscicidal, and repellent activities [2,4]. As for flavor, research has focused on processing methods for extracting ZA oil, identifying its key flavor compounds, and comparing flavor differences between ZA and *Zanthoxylum bungeanum* Maxim (ZB). Hu et al. found that ZA oil extract via ultrasound-assisted extraction exhibits the highest levels of aromatic and numbing components, as well as superior stability [5]. Meanwhile, the high-temperature frying method is easy to use and relatively low-cost. It uses high temperature to rupture the cell walls of ZA, releasing aromas that dissolve into the vegetable oil [6]. Linalool, D-limonene, sabinene, myrcene, *β*-phellandrene, and geraniol and linalyl acetate were identified as the main odorants in ZA oil [5,7,8]. Specifically, linalool, sabinene, linalyl acetate, and piperitone were markers for distinguishing between ZA oil samples and ZB samples [8,9]. Among *α*-, *β*-, *γ*-, and *δ*-sanshool and their derivatives, it was found that hydroxyl-*α*-sanshool significantly enhances the phenomenon of a numbing sensation in ZA [10,11,12]. There is no doubt that the difference between fresh (FFTO) and dried (DFTO) fried *Tengjiao* oil might be related to the *Tengjiao* drying process. During the drying process of Sichuan pepper, alcohols may be converted into terpenes, resulting in a decrease in alcohol content and an increase in terpene content [13]. The drying method is a key factor affecting the volatile compounds in ZA and ZB. In particular, hot-air drying at lower temperatures helps preserve its key flavor compounds [14]. In fried *Tengjiao* oil (FTO), the frying process significantly transforms the composition and proportions of flavor compounds through thermal degradation, oxidative reactions, and interactions with the oil medium. It is found that frying time and temperature have a significant impact on flavor quality [15,16]. For example, high temperatures might accelerate the thermal degradation, thermal oxidation, and isomerization of linalool and other compounds [17]. However, the flavor differences between FFTO and DFTO, as well as the reasons for these flavor differences, have not yet been systematically studied by scholars.

Molecular sensory science is currently the dominant and systematic approach for analyzing the flavor of food. It has been developed by Peter Schieberle’s research team and has a history of approximately 18 years [18]. It is a multidisciplinary technology for researching food flavor quality at the molecular level. It employs chromatography, mass spectrometry, and sensory evaluation techniques, combined with olfactory assessment and odor activity value (OAV) to screen aroma compounds, and validates key flavor compounds through recombination and omission experiments [19]. Molecular sensory science has been applied in the flavor analysis of ZA and ZB. For example, Sun et al. identified *β*-phellandrene, *p*-cymene, acetic acid octyl ester, octanal, citronellol, and sabinene as the major factors responsible for the flavor differences between Hancheng and Hanyuan fried ZB oils through molecular sensory science technology [20]. Additionally, linalool, linalyl acetate, and 1,8-cineole have been identified as flavor difference markers for ZB oils and *Zanthoxylum schinifolium* sieb. et Zucc. (ZS) oils using molecular sensory science technology [21]. It is noteworthy that in nature, the chiral structure of flavor components influences the overall flavor profile of food. For example, the odor profiles of different menthol isomers exhibit significant differences [22]. Previous studies have examined the enantiomeric ratio of limonene and linalool in fried ZB oil, revealing that S-(−)-limonene and S-(+)-linalool align more closely with the aromatic attributes of these compounds in fried ZB oil [20]. However, the effect of enantiomeric ratios of terpenoids in FTO and the differences between FFTO and DFTO enantiomers on flavor has not yet been studied.

In this study, volatile organic compounds (VOCs) in FFTO and DFTO were extracted via solvent-assisted flavor evaporation (SAFE). Then, gas chromatograph–mass spectrometry (GC-MS) and gas chromatography–olfactometry–mass spectrometry (GC-O-MS) combined with aroma extract concentration analysis (AEDA) were employed for the qualitative and quantitative analysis of volatile flavor compounds in FTO. Subsequently, odor activity values (OAVs) were calculated to determine the contribution of each VOC to the overall aroma of the FTO samples. After that, recombination and omission experiments were performed to identify and validate key flavor compounds. In particular, the enantiomers of limonene and linalool in FTOs were detected to determine their respective contributions to the flavor profile of FTOs.

## 2. Materials and Methods

### 2.1. Materials

Hongya Tengjiao samples (from Hongya County, Meishan, China) were obtained from Chengdu Zhuojia Trading Co., Ltd. (Chengdu, China). Rapeseed oil was obtained from Yihai Kerry Grain and Oil Co., Ltd. (Chongqing, China).

### 2.2. Chemical Standards

Linalyl acetate (95%) was purchased from Acros Organics (Shanghai, China). Germacrene D (>90%), *γ*-terpinene (>95%), 5-hexenenitrile (>95%), 3-buten-1-yl isothiocyanate (>96%), *cis*-linalool oxide (>97%), 4-octanol (>97%), 2-methylpyrazine (>98%), ethylbenzene (>99.7%), thujone (≥70%), (*E*,*E*)-2,4-decadienal (≥97%), 4-pentenoic acid (≥98%), *o*-xylene (≥99%), *p*-xylene (≥99.8%), *β*-myrcene (90%), terpinen-4-ol (95%), camphene (95%), 3-phenylpropionitrile (97%), 3-butenenitrile (97%), styrene (99%), 2-acetylfuran (99%), and methyl phenylacetate (99%) were purchased from Aladdin (Shanghai, China). (*E*)-2-Octenal was purchased from Alfa Aesar (Shanghai, China). 4-Octanone (98%), amylene hydrate (99%), furfuryl alcohol (99%) and phenylethyl alcohol (99%) were purchased from Adamas-beta (Shanghai, China). *β*-Pinene (>95%), 2(5H)-furanone (95%), linalool (98%), and 1-butanol (99.5%) were purchased from Innochem (Beijing, China). (*E*,*E*)-2,4-Heptadienal (90%), (*E*)-2-heptenal (95%), 2-acetyl pyrrole (98%), 1-penten-3-ol (98%), 2-pentyl-furan (98%), phenethyl acetate (98%), 2,5-dimethyl pyrazine (99%), methyl cyclopentenolone (99%), butyric acid (99%), and acetic acid (99.8%) were purchased from J&K Scientific (Beijing, China). *α*-Phellandrene (≥85%), 1-propanol (≥99.8%), *α*-caryophyllene (93%), myrtenol (95%), *trans*-*β*-ocimene (95%), *β*-Phellandrene (98%), 3-ethyl-2,5-dimethylpyrazine (98%), and pentanal (98%) were purchased from Macklin (Beijing, China). Dichloromethane (99.5%), 5-methyl furfural (>98%), *α*-pinene (98%), *β*-caryophyllene (98%), and eucalyptol (99%) were purchased from Mreda (Beijing, China). n-Alkanes (C_6_–C_30_) and *p*-cymene (99%) were purchased from Sigma-Aldrich (Shanghai, China). Indole (>90%), *α*-terpinene (>90%), terpinolene (>90%), *trans*,*trans*-2,4-decadienal (>90%), piperitone (>94%), *trans*-2-pentenal (>95%), limonene (>95%), heptanal (>95%), nonanal (>95%), (*E*)-2-hexenal (>95%), hexanal (>98%), octanal (>98%), furfural (>98%), citronellal (>98%), N-methyl-2-pyrrolidone (>99%), *γ*-butyrolactone (>99%), and benzaldehyde (98%) were purchased from TCI (Shanghai, China). 2-Hydroxy-3-Methyl-2-cyclopentenone (98%) was purchased from Energy Chemical (Shanghai, China). *α*-Terpineol (≥96%), *α*-thujone (≥98%), and sabinene (≥98%) were purchased from Yuanye (Shanghai, China).

### 2.3. FTO Sample Preparation

Preparation of dried *Tengjiao*: Fresh *Tengjiao* (80 g) fruits were weighed and placed in an odorless, uncovered glass Petri dish. Subsequently, they were dried in a Non-Standard Type 10 Electric Heating Dryer Control System (Changzhou Erle Drying Equipment Co., Ltd., Changzhou, China) for 10 h (55–65 °C, 1 m/s).

Preparation of FTO Samples: Our group has conducted in-depth research into the optimal processing conditions for frying pepper oil. We referred to the method that our group previously used to prepare fried pepper oil, with slight modifications [15,23]: 150 g of rapeseed oil was poured into a 500 mL glass flask, which was then placed in an oil bath for heating while the temperature of the rapeseed oil was monitored. At the same time, magnetic stirring was carried out to ensure even heating. When the oil temperature stabilized at 130 °C, the *Tengjiao* fruit (80 g, in fresh *Tengjiao* quality) was added to the oil, and frying began. When the frying time reached 20 min, the flask was quickly removed and the heating process was stopped. The *Tengjiao* fruits were then filtered out, and the FTO was cooled in an ice-water bath. The prepared FTO samples were set aside in a −40 °C refrigerator. All samples were prepared in triplicate for subsequent determination experiments.

### 2.4. Quantitative Descriptive Analysis (QDA)

The sensory evaluation panel was assembled from members of the Beijing Key Laboratory of Flavor Chemistry at Beijing Technology and Business University. The panel comprised 12 panelists, including 5 males and 7 females, all between the ages of 20 and 30. The odor of the experimental samples posed no danger or discomfort to the participants. Furthermore, the participants signed written consent forms after being fully informed of the details, and they had the right to withdraw from the experiment at any time. The sensory evaluations were conducted in a specialized sensory evaluation room maintained at 25 °C and free of odors. Before the formal experiment began, the sensory panel underwent professional training. This sensory evaluation experiment was approved under Certificate No. 139 of 2025 issued by the Scientific Research Ethics Committee of Beijing Technology and Business University.

Firstly, 2 g of FTO was added to a 10 mL, odorless, transparent glass vial with a cap. Based on the participants’ discussions regarding the flavor characteristics of FFTO and DFTO, the aroma descriptors for FTO were determined based on the frequency of occurrence of the descriptors. Ultimately, a total of eight descriptors—oil, pepper, spice, roasted, herbal, wood, citrus-like, and green notes—were identified. The panelists were required to rate the intensity of each descriptor on a 10-point linear scale ranging from 0 to 9 (from completely imperceptible to strongly perceptible).

### 2.5. Isolation of Volatiles by SAFE

FTO (50 g) was placed in a separating funnel (500 mL), followed by CH_2_Cl_2_ solvent (200 mL) and internal standard (4-octanol, 82.2 mg/mL, 20 μL) for extraction. Liquid–liquid extraction was carried out using a GGC-C separating funnel vertical shaker (Beijing Guohuan High-tech Automation Technology Research Institute, Beijing, China) at 300 r/min for 20 min. Then, the sample was transferred to a SAFE dropping funnel for high-vacuum distillation. When the pressure of the whole device dropped to 1 × 10^−5^ mbar, we slowly rotated the piston of the dropping funnel to start distillation. After the distillation was essentially complete, an empty distillation was performed for 40 min to fully extract the volatile compounds from FTO. After that, FTO volatile component extracts were obtained. After the temperature of the extract was stabilized to room temperature, the appropriate amount of anhydrous Na_2_SO_4_ was added to remove the water. The final distillate was concentrated to 1 mL using a Vigreux column (50 cm × 1 cm) (Beijing Jingxing Glassware Co., Ltd., Beijing, China) and BF-2000 nitrogen drying instrument (Beijing Bafang Century Technology Co., Ltd., Beijing, China). The concentrate was stored in a −40 °C freezer for later detection.

### 2.6. GC-MS Analysis

GC-MS analysis was performed using a Thermo Fisher Trace 1300 gas chromatograph (Thermo Fisher Scientific, Waltham, MA, USA) combined with a Thermo Fisher mass spectrometer (Thermo Fisher Scientific, Waltham, MA, USA). Separation was performed using a TG-Wax column (30 m × 0.25 mm i.d., 0.25 μm, Thermo Fisher Scientific, Waltham, MA, USA).

The program referred to the method of Sun et al., with slight modifications [20]. Helium was used as the carrier gas and delivered at a fixed flow rate of 1.2 mL/min to the column. For the TG-Wax column, the oven temperature was initially 40 °C, followed by a 1 min hold; increased to 140 °C at a rate of 2 °C/min, followed by a 1 min hold; and finally increased to 220 °C at a rate of 6 °C/min, followed by a 1 min hold. The mass detector conditions were as follows: ionization energy, 70 eV; MS transfer line temperature, 230 °C; ion source temperature, 250 °C; mass range, *m*/*z* 40–350; and solvent delay, 4 min.

### 2.7. GC-O-MS

GC-O-MS analysis was achieved using a GC-MS (7890B Gas Chromatography System equipped with 5977B GC/MSD (Agilent Technologies Inc., Santa Clara, CA, USA)) with an olfactometer detector (ODP 3; Gerstel, Mulheim an der Ruhr, Germany). The GC-MS analysis conditions were consistent with those described in Section 2.6. For olfactometer detector, the transmission line temperature was 250 °C and the exit temperature was 120 °C. During the analysis, these flavor compounds were split at a 1:1 ratio via a three-way valve and directed into the mass spectrometry detector and the olfactory interface. In particular, during the olfactory experiments, a continuous supply of moist air was used to prevent the participants’ nasal passages from drying out. In addition, a GC-O-MS evaluation panel consisting of three experts selected from the panel in Section 2.4 recorded the time, intensity, and characteristics of each odor. The three experts showed qualities such as strong concentration, a keen sense of smell, and familiarity with and sensitivity to the odor characteristics of FTO. During the data verification phase, each compound had to be identified by at least two experts before it could be deemed to be an aroma-active compound.

We combined aroma extract concentration analysis (AEDA) with GC-O-MS to investigate the sensory characteristics and intensity of various volatile compounds in FTO. The flavor dilution (FD) factor for the highest concentration sample (undiluted FTO concentrate) was designated as 1. Serial dilutions were performed at a ratio of V (undiluted FTO concentrate):V (dichloromethane) = 1:3. Each dilution was subjected to an olfactory assessment, with the detected odors and corresponding retention times recorded. When the dilution ratio reached a level where the odor could no longer be detected by smell, the process could be stopped. The above analysis results were expressed as FD factors.

### 2.8. Qualitative and Quantitative Analysis

The volatile compounds in FTOs were identified using the NIST 17 library (MS) retention index (RI) combined with standards (Std). First, semi-quantitative analysis was applied to compounds identified by GC-MS. For compounds with FD ≥ 27 in the AEDA process, precise quantification was performed by an internal standard curve method based on semi-quantitative results.

### 2.9. OAV Analysis

In molecular sensory science and technology, OAV is one of the key indexes for evaluating the contribution of various volatile compounds to the overall aroma of a sample. OAV is defined as the ratio of the concentration of a substance to its odor threshold in water or oil [24]. It is calculated by the formula OAV_i_ = c_i_/OT_i_; among them, OAV_i_ is the aroma activity value of each ingredient, Ci is the content of each aroma component in the sample (mg/kg), and OT_i_ is the aroma threshold value of each ingredient (mg/kg). The thresholds in this study were obtained from *Odor thresholds* [25].

### 2.10. Recombination and Omission Experiments

Before the recombination experiment, we first prepared an odorless matrix as the solvent. For the preparation method for the odorless matrix, we referred to the previous program used by our group, with minor modifications [23]. The rapeseed oil was heated in an oil bath at 130 °C for 20 min. After the rapeseed oil had cooled to room temperature, it was first mixed with 100 mL of dichloromethane for liquid–liquid extraction (30 min) to remove the flavor compounds from the rapeseed oil. Dichloromethane was then removed by rotary evaporation, and extraction was continued with 100 mL of n-pentane. The above process was repeated three times. Finally, the extracted rapeseed oil was transferred into an open beaker, and the residual solvent was removed using a magnetic stirrer (IKA RH D S025) at 60 °C, 300 r.

Based on the quantitative results, aroma recombination for FFTO and DFTO was performed. Aroma compounds with an OAV > 1 were used and added to the odorless FPO matrix. The recombinant samples were evaluated using the attributes employed in sensory evaluation. After this, a triangle test was implemented to verify the significant differences between the omitted and recombinant samples.

### 2.11. Detection of Enantiomers

Analysis of the enantiomers was performed using a GC-MS (Thermo Fisher Trace 1310 gas chromatograph (Thermo Fisher Scientific, Waltham, MA, USA) combined with a Thermo Fisher mass spectrometer (Thermo Fisher Scientific, Waltham, MA, USA). Separation was performed using a BGB-176SE column (30 m × 0.25 mm i.d., 0.25 μm, BGB-Analytik AG, Switzerland). Helium was used as the carrier gas and delivered at a fixed flow rate of 2 mL/min to the column. Other chromatographic and mass spectrometry conditions were the same as for 2.4 GC-MS. Qualitative analysis was performed using mass spectrometry (MS) and standards (S), while quantitative analysis was conducted using semi-quantitative methods.

### 2.12. Statistical Analysis

Experimental data were collected and organized using Excel (Microsoft Office 2016, Redmond, WA, USA). The results of the experiments were expressed as the mean of three experiments ± standard deviation. The *t*-test function was used to analyze significant differences using Excel (Microsoft Office 2016, Redmond, WA, USA). Aroma profile, bar chart and Venn diagram were plotted using Origin version 2025b (OriginLab Corporation, Northampton, MA, USA). PLSR analysis was conducted using XLSTAT version 2019 (Addinsoft, New York, NY, USA). Heat map was created by TBtools-II 2.081software (https://github.com/CJ-Chen/TBtools, accessed on 28 November 2025).

## 3. Results and Discussion

### 3.1. Sensory Profiles of DFTO and FFTO

**Figure 1:**
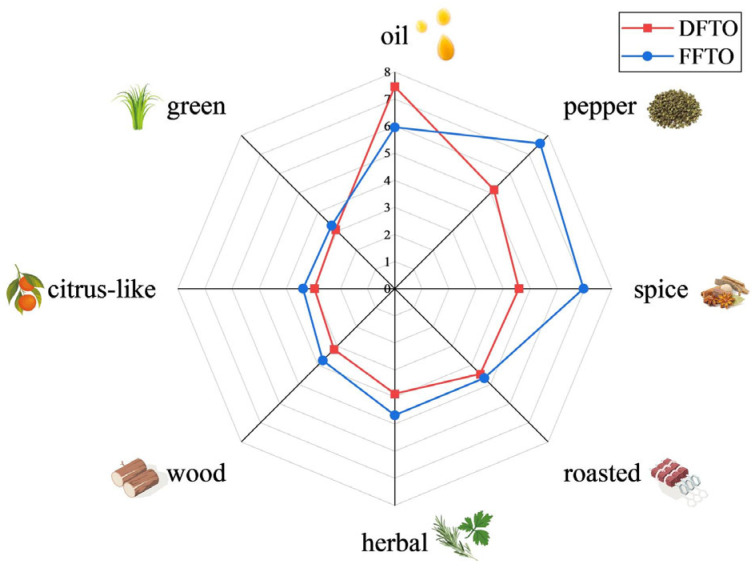
Aroma profiles of DFTO and FFTO by sensory evaluation. (Each of the 12 participants completed one evaluation. A *t*-test was performed. Oil, pepper, and spice: *p* < 0.05. FFTO, fresh fried *Tengjiao* oil; DFTO, dried fried *Tengjiao* oil.)

In order to illustrate the flavor differences between FFTO and DFTO more clearly, the aroma profiles are presented using a radar chart (Figure 1). Based on evaluation and screening by sensory panels, eight aroma attributes were used to describe the flavor characteristics of FTOs. They were oil, pepper, spice, roasted, herbal, wood, citrus-like, and green. FFTO and DFTO had similar sensory attributes, but the intensity of each attribute was different, resulting in distinct flavors. The intensity of pepper aroma and spice aroma in FFTO (pepper, 7.58; spice, 6.96) was significantly higher than that in DFTO (pepper, 5.17; spice, 4.58). Meanwhile, the intensity of the oil aroma in DFTO (7.46) was obviously higher than that in FFTO (5.96). The intensity of other aroma attributes (roasted, herbal, wood, citrus-like, and green) showed minimal variation between FFTO and DFTO. It is worth noting that the intensity of these aroma attributes was slightly higher in FFTO than in DFTO. Overall, FFTO exhibited a higher overall aroma intensity than DFTO, particularly in terms of the pepper and spice aroma. The cause of this result might be the loss or transformation of some volatile compounds during the drying process or the frying process of *Tengjiao*.

### 3.2. Identification of Volatile Compounds in FTOs by GC-MS

**Figure 2:**
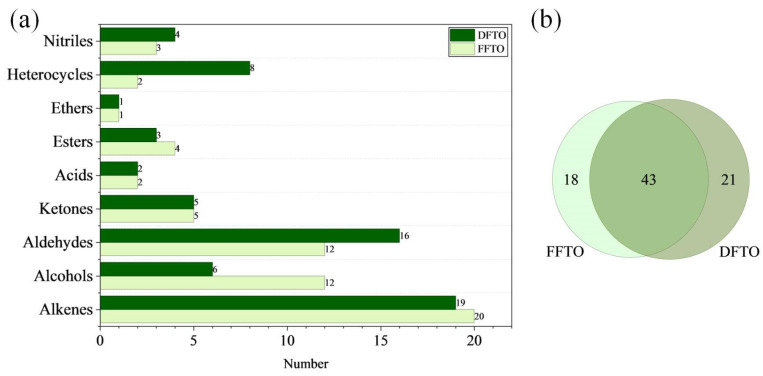
Comparison of volatile compounds in FFTO and DFTO. (**a**) Classification and number of volatile compounds in FTOs. (**b**) Venn diagram of FFTO and DFTO volatile compounds. (FFTO, fresh fried *Tengjiao* oil; DFTO, dried fried *Tengjiao* oil).

A total of 82 volatile compounds (22 alkenes, 13 alcohols, 18 aldehydes, 7 ketones, 3 acids, 5 esters, 1 ether, 9 heterocycles, and 4 nitriles) were identified in FTOs (Table A1). The number of volatile compounds in FFTO and DFTO was not significantly different, with 61 and 64 compounds, respectively. Among them, they shared as high as 43 volatile compounds (Figure 2a). These compounds might contribute to the characteristic flavor profile of FTO, such as its pepper and spice aroma. However, 18 volatile compounds were unique to FFTO, while 21 volatile compounds were unique to DFTO. These compounds might be the main reason for the flavor distinctions between FFTO and DFTO. In terms of compound types, most types of compounds did not differ significantly (alkenes, ketones, acids, esters, ether, and nitriles). Notably, FFTO contained a greater variety of alcohols, while DFTO contained a greater variety of aldehydes and heterocyclic compounds (Figure 2b). This might be due to the relatively high content of terpenols in fresh *Tengjiao* berries, which undergo transformation and degradation during the drying process [13]. Moreover, the drying process also resulted in the formation of more aldehydes and heterocyclic compounds in *Tengjiao* [26]. It is noteworthy that only two heterocyclic compounds were present in FFTO, whereas eight were found in DFTO, with seven of them newly generated. They were 2-pentyl-furan, 2-methylpyrazine, 2,5-dimethyl pyrazine, 3-ethyl-2,5-dimethylpyrazine, 2-acetylfuran, N-methyl-2-pyrrolidone, 2(5H)-furanone, and 2-acetyl pyrrole. In this case, pyrazine and pyrrole might originate from the Maillard reaction [27,28], while furan could come from lipid oxidation [29].

In terms of the number of various compounds, alkenes, aldehydes, and alcohols were more abundant in FTOs. The abundance of alkenes, alcohols, and aldehydes was consistent with previous studies [1]. The alkenes and alcohols may mainly originate from *Tengjiao*, while the aldehydes may primarily come from rapeseed oil [30]. Among these, terpenes might give FTO its characteristic floral (linalool, etc.), woody (caryophyllene, etc.), citrus (limonene, etc.) and other flavors. Meanwhile, aldehydes might impart FTO with rich green (hexanal, etc.) and oily (2,4-decadienal, etc.) aromas. In summary, based on the number of compounds, alkenes, aldehydes, and alcohols played a key role in the formation of FTO flavor. Heterocyclic compounds, alcohols, and aldehydes may be the primary factors causing the flavor differences between FFTO and DFTO. This conclusion still required validation through further quantitative experiments.

### 3.3. Aroma Intensity of Each Compound Determined by AEDA

**Figure 3:**
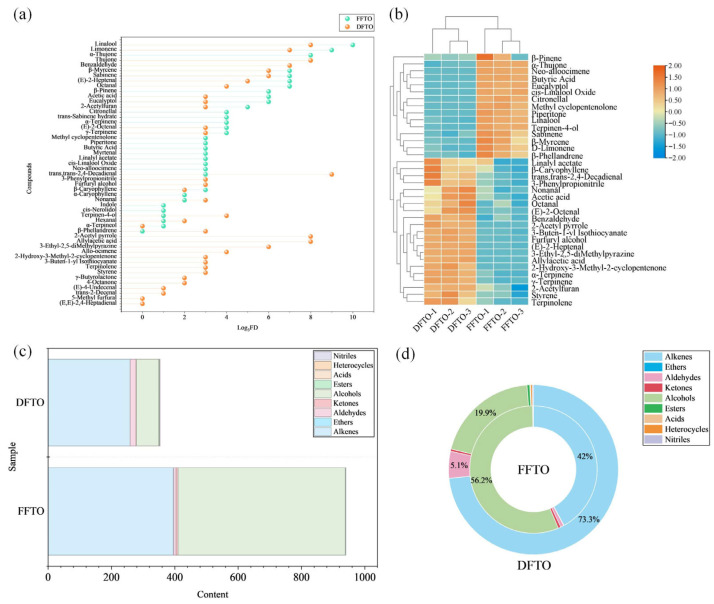
Comparison of aroma-active compounds in FFTO and DFTO. (**a**) FD factors of aroma-active compounds in FTOs. (**b**) Content of aroma-active compounds in FTOs (FD ≥ 3^3^). (**c**) Comparison of aroma-active compound content in FTOs (FD ≥ 3^3^). (**d**) Percentage distribution of various aroma-active compounds. (FFTO, fresh fried *Tengjiao* oil; DFTO, dried fried *Tengjiao* oil).

In order to determine whether volatile compounds in FTOs have aroma activity and consequently influence the flavor of FTOs, we performed an analysis by GC-O-MS combined with AEDA. In FFTO and DFTO, there were 44 and 49 aroma-active compounds, respectively (Table 1). A total of 37 and 36 aroma-active compounds were successfully identified through MS, RI or S qualification, respectively (Figure 3a).

In FFTO, there was 1 compound with FD = 3^10^ (linalool), 1 compound with FD = 3^9^ (limonene), 2 compounds with FD = 3^8^ (thujone and *α*-thujone), 5 compounds with FD = 3^7^ (sabinene, *β*-myrcene, octanal, (*E*)-2-heptenal, and benzaldehyde), 3 compounds with FD = 3^6^ (*β*-pinene, eucalyptol, and acetic acid), 1 compound with FD = 3^5^ (2-acetylfuran), 5 compounds with FD = 3^4^ (*α*-terpinene, *γ*-terpinene, (*E*)-2-octenal, *trans*-sabinene hydrate, and citronellal), and 11 compounds with FD = 3^3^ (neo-alloocimene, *cis*-linalool oxide, linalyl acetate, *β*-caryophyllene, myrtenal, butyric acid, furfuryl alcohol, piperitone, *trans*,*trans*-2,4-decadienal, methyl cyclopentenolone, and 3-phenylpropionitrile). Additionally, six unknown compounds had FD = 3^3^. These aroma-active compounds might contribute to flavor formation in FFTO. Linalool had the highest FD factor and exhibited floral, lavender, lemon, and sweet aromas. It has been demonstrated to be a key flavor compound in Sichuan pepper and related products across a multitude of studies [20,31,32]. Limonene has the second-highest FD factor, exhibiting citrus and herb flavor characteristics. It has also been demonstrated to be one of the key flavor compounds in Sichuan peppers and related products [20,33]. It exhibits extremely high aroma intensity in FFTO, but its contribution to the overall flavor profile requires further verification.

In DFTO, there was 1 compound with FD = 3^9^ (*trans*,*trans*-2,4-decadienal), 4 compounds with FD = 3^8^ (thujone, linalool, allylacetic acid and 2-acetyl pyrrole), 2 compounds with FD = 3^7^ (limonene and benzaldehyde), 2 compounds with FD = 3^6^ (sabinene, *β*-myrcene and 3-ethyl-2,5-dimethylpyrazine), 1 compound with FD = 3^5^ ((*E*)-2-heptenal), 3 compounds with FD = 3^4^ (octanal, allo-ocimene and terpinen-4-ol), and 13 compounds with FD = 3^3^ (eucalyptol, *β*-phellandrene, *γ*-terpinene, styrene, terpinolene, nonanal, (*E*)-2-octenal, 3-buten-1-yl isothiocyanate, acetic acid, 2-acetylfuran, furfuryl alcohol, 2-hydroxy-3-methyl-2-cyclopentenone, and 3-phenylpropionitrile). In addition, two unknown compounds had FD = 3^8^, one unknown compound had FD = 3^6^, two unknown compounds had FD = 3^5^, three unknown compounds had FD = 3^4^, and three unknown compounds had FD = 3^3^. These aroma-active compounds might contribute to flavor formation in DFTO. *trans*,*trans*-2,4-Decadienal was the volatile compound with the highest FD factor in DFTO, exhibiting characteristic fat, oil, and pepper odors. It most likely originated from rapeseed oil. It has been shown in previous research to be an important product of the thermal oxidation of linoleic acid [29]. Thujone exhibited a green, cucumber-like odor, allylacetic acid exhibited a fruity sweet aroma, and 2-acetyl pyrrole exhibited a roasted aroma. From the perspective of aroma intensity, they might contribute to flavor formation in DFTO.

In particular, aroma-active compounds such as linalool, limonene, and thujone showed higher FD factors in both FTO samples, potentially making significant contributions to the flavor of FTOs. In summary, 29 and 27 aroma-active compounds with FD > 3^3^ were successfully identified in FFTO and DFTO, respectively, and they may be closely related to the flavor of FTOs. Of course, this still requires verification through further analysis and experimentation.

### 3.4. Content of Aroma-Active Compounds

To precisely determine the content of each aroma-active compound, we employed the standard curve method to quantify 36 aroma-active compounds (FD ≥ 3^3^) that had already been qualitatively identified using standards (Table 2). Based on the *t*-test results for each aroma-active compound, 20 compounds showed significant differences between FFTO and DFTO (*p* < 0.05). These compounds may contribute to the flavor profile differences between FFTO and DFTO. To show the differences in the content of FFTO and DFTO aroma-active compounds more intuitively, we created a heat map (Figure 3b). The heat map clearly demonstrates that there are significant differences between FFTO and DFTO. We can obviously divide the heat map into two parts. In the upper half, 15 compounds (*β*-pinene to *β*-phellandrene) have higher concentrations in FFTO, while in the lower half, 21 compounds (linalyl acetate to terpinolene) have higher concentrations in DFTO. This result might be due to the degradation and transformation of volatile compounds during the drying process of the *Tengjiao* fruit. In terms of the total amount of aroma-active compounds, the content in FFTO was significantly higher than that in DFTO, with FFTO content even exceeding that of DFTO by more than two times (Figure 3c). Both FTO samples contained abundant alkenes and alcohols. Notably, the alcohol and alkene contents were significantly reduced in the DFTO. In FFTO, the most abundant aroma-active compounds were alcohols (56.2%) and alkenes (42%), whereas in DFTO, the most abundant aroma-active compounds were alkenes (73.3%), alcohols (19.9%), and aldehydes (5.1%) (Figure 3d). In summary, FFTO and DFTO shared similar types of aroma-active compounds, but exhibited significant differences in the content of each component. This difference in content might be the underlying reason for the difference in flavor between the two.

**Table 2:** The results of OAV for FFTO and DFTO.

No.	Compound	Standard Curve	R^2^	Content/(μg/g)		Odor Thresholds/ (μg/g)	OAV	
				FFTO	DFTO		FFTO	DFTO
**1**	*β*-Pinene	y = 0.9756x − 0.0067	0.9965	3.307 ± 0.464	2.926 ± 0.031	1.5	2.20	1.95
**2**	Sabinene	y = 0.6253x + 0.1179	0.9945	140.049 ± 17.954	75.993 ± 4.924	0.98	142.91	77.54
**3**	*β*-Myrcene	y = 1.0413x − 0.0245	0.9967	19.607 ± 1.573	15.209 ± 0.146	0.915	21.43	16.62
**4**	*α*-Terpinene *	y = 0.907x − 0.0014	0.995	1.095 ± 0.201	3.553 ± 0.236	0.085	12.88	41.80
**5**	Limonene	y = 0.5897x + 0.0358	0.9981	209.395 ± 18.876	139.328 ± 5.053	1.2	174.50	116.11
**6**	Eucalyptol *	y = 0.4856x + 0.0006	0.9954	0.834 ± 0.035	0.065 ± 0.009	0.015	55.60	4.33
**7**	*β*-Phellandrene	y = 0.7298x − 0.0088	0.9948	18.256 ± 1.53	13.289 ± 0.384	0.036	507.11	369.14
**8**	*γ*-Terpinene *	y = 0.8966x − 0.0007	0.992	1.87 ± 0.18	5.754 ± 0.447	2.89	0.65	1.99
**9**	Styrene *	y = 0.8342x + 0.0003	0.9961	0.341 ± 0.1	0.841 ± 0.124	3.1	0.11	0.27
**10**	Terpinolene	y = 0.9671x − 0.0047	0.9941	0.947 ± 0.066	1.436 ± 0.153	0.2	4.74	7.18
**11**	Octanal	y = 0.2807x − 0.0001	0.9924	0.358 ± 0.088	0.762 ± 0.145	0.32	1.12	2.38
**12**	(*E*)-2-Heptenal *	y = 0.2824x − 0.0008	0.9987	1.005 ± 0.076	5.564 ± 0.144	14	0.07	0.40
**13**	Neo-alloocimene *	y = 2.6262x − 0.0019	0.9924	0.027 ± 0.001	-	-	-	-
**14**	Nonanal	y = 0.0934x − 0.003	0.9912	5.491 ± 0.867	10.052 ± 2.408	13.5	0.41	0.74
**15**	*α*-Thujone *	y = 0.4749x − 0.0004	0.9997	5.69 ± 0.309	0.606 ± 0.138	0.36	15.81	1.68
**16**	(*E*)-2-Octenal	y = 0.3507x − 0.0002	0.9919	0.148 ± 0.015	0.418 ± 0.094	7	0.02	0.06
**17**	3-Ethyl-2,5-diMethylpyrazine *	y = 0.7952x − 0.0004	0.9991	-	0.228 ± 0.004	0.024	-	9.50
**18**	*cis*-Linalool oxide *	y = 0.2468x + 0.0001	0.9971	0.792 ± 0.044	-	6	0.13	-
**19**	3-Buten-1-yl Isothiocyanate *	y = 0.6187x − 0.0002	0.9953	0.134 ± 0.011	0.38 ± 0.017	0.017	7.88	22.35
**20**	Acetic acid	y = 0.6379x − 0.0021	0.9933	0.18 ± 0.046	0.379 ± 0.078	0.5	0.36	0.76
**21**	Citronellal *	y = 0.3727x − 0.0003	0.9995	0.295 ± 0.019	-	0.046	6.41	-
**22**	2-Acetylfuran	y = 0.6467x + 0.0004	0.9961	0.163 ± 0.117	0.465 ± 0.01	10	0.02	0.05
**23**	Benzaldehyde *	y = 0.7671x − 0.0005	0.9994	0.091 ± 0.016	0.183 ± 0.008	0.06	1.52	3.05
**24**	Linalool *	y = 0.4882x + 0.3236	0.9944	520.354 ± 68.832	68.403 ± 8.984	0.037	14,063.62	1848.73
**25**	Linalyl acetate	y = 0.2163x − 0.0002	0.991	1.099 ± 0.315	1.513 ± 0.218	1	1.10	1.51
**26**	*β*-Caryophyllene	y = 0.3392x − 0.0005	0.9943	0.496 ± 0.109	0.815 ± 0.128	0.064	7.75	12.73
**27**	Terpinen-4-ol *	y = 0.4935x − 0.0005	0.9993	6.576 ± 0.746	0.487 ± 0.051	0.59	11.15	0.83
**28**	Butyric acid *	y = 0.9269x − 0.0006	0.9961	0.025 ± 0.001	-	0.109	0.23	-
**29**	Furfuryl alcohol *	y = 0.2295x + 0.0001	0.9975	0.546 ± 0.019	1.466 ± 0.048	1.9	0.29	0.77
**30**	Piperitone *	y = 0.5743x − 0.0007	0.9962	0.268 ± 0.031	-	0.68	0.39	-
**31**	*trans*,*trans*-2,4-Decadienal	y = 0.3071x − 0.001	0.9912	0.587 ± 0.079	1.139 ± 0.213	2.5	0.23	0.46
**32**	Allylacetic acid *	y = 0.5277x − 0.0014	0.9937	-	0.11 ± 0.006	-	-	-
**33**	2-Hydroxy-3-Methyl-2-cyclopentenone	y = 0.2072x − 0.0003	0.9959	-	0.957 ± 0.177	2	-	0.48
**34**	Methyl cyclopentenolone *	y = 0.1751x − 0.0002	0.9982	0.345 ± 0.036	-	2	0.17	-
**35**	2-Acetyl pyrrole *	y = 0.4903x + 1 × 10^−5^	0.999	-	0.425 ± 0.039	58.58525	-	0.01
**36**	3-Phenylpropionitrile	y = 0.748x − 0.0001	0.9996	0.398 ± 0.068	0.852 ± 0.132	0.015	26.53	56.80

“-” indicates that the compound was not detected or the odor threshold was not found. “*” indicates that *p* < 0.05 in the *t*-test, indicating a statistically significant difference. The contents are expressed as the average of three experiments, presented as the mean ± standard deviation. (FFTO, fresh fried *Tengjiao* oil; DFTO, dried fried *Tengjiao* oil; OAV, odor activity value).

### 3.5. Correlation Between Major Aroma-Active Compounds and Aroma Attributes

**Figure 4:**
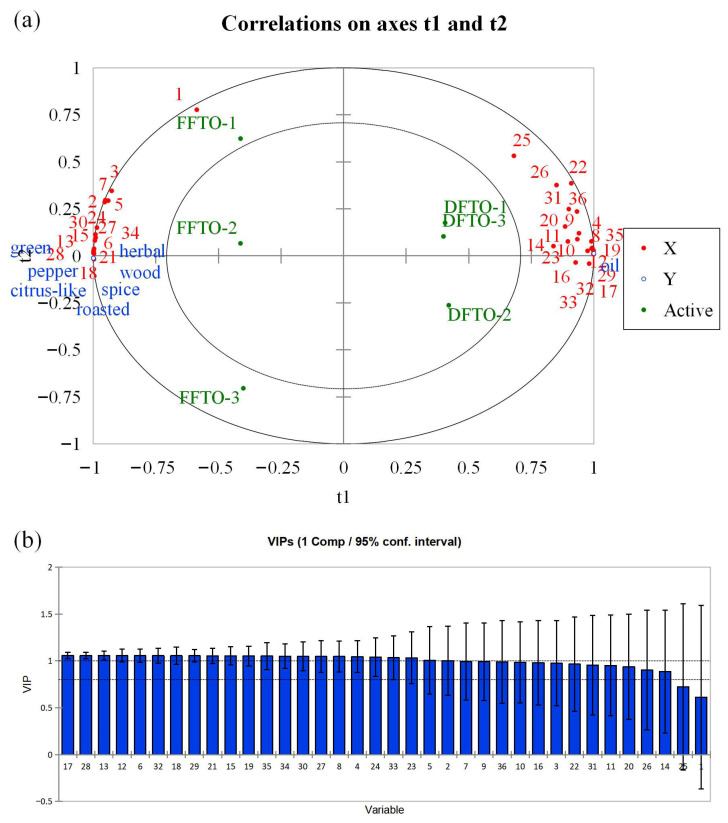
Correlation analysis of FTOs by partial least squares regression (PLSR). (**a**) Correlation loadings of the PLSR model between sensory attributes and aroma-active compounds in fried *Tengjiao* oils (FTOs). (**b**) Variable importance for the projection (VIP) scores and 95% confidence intervals (bootstrap method) of aroma-active compounds for the first component in the PLSR model.

To further investigate the relationship between aroma-active compounds and aroma attributes in FFTO and DFTO, we performed partial least squares regression (PLSR) analysis. The X-matrix was designed to represent the aroma-active compounds in FTOs, while the Y-matrix was designed for sensory attributes. As shown in Figure 4a, most of the X-matrix and Y-matrix are located around the circle r^2^ = 1, which indicates that the PLSR model can adequately explain the above variables [34]. FFTO and DFTO exhibit distinct differences, distributed in the second and third quadrants and the first and fourth quadrants, respectively. Additionally, this figure also provides clearer sensory evaluation results: FFTO exhibits more pronounced notes of green, pepper, spice, roasted, herbal, wood, and citrus-like aromas, while DFTO displays a more pronounced oil aroma. Specifically, compounds 1 (*β*-pinene), 3 (*β*-myrcene), 7 (*β*-phellandrene), 2 (sabinene), 5 (limonene), 24 (linalool), 30 (piperitone), 27 (terpinen-4-ol), 15 (*α*-thujone), 34 (methyl cyclopentenolone), 13 (neo-alloocimene), 6 (eucalyptol), 28 (butyric acid), 21 (citronellal), and 18 (*cis*-linalool oxide) show a significant positive correlation with FFTO, while compounds 25 (linalyl acetate), 26 (*β*-caryophyllene), 22 (2-acetylfuran), 31 (*trans*,*trans*-2,4-decadienal), 36 (3-phenylpropionitrile), 20 (acetic acid), 9 (styrene), 11 (octanal), 4 (*α*-terpinene), 14 (nonanal), 23 (benzaldehyde), 10 (terpinolene), 8 (*γ*-terpinene), 35 (2-acetyl pyrrole), 19 (3-buten-1-yl isothiocyanate), 12 ((*E*)-2-heptenal), 16 ((*E*)-2-octenal), 29 (furfuryl alcohol), 32 (allylacetic acid), 33 (2-hydroxy-3-methyl-2-cyclopentenone), and 17 (3-ethyl-2,5-dimethylpyrazine) show significant positive correlations with DFTO. Visually, the active compounds in quadrants 1 and 4 may be related to oil aroma. However, the remaining aroma attributes are clustered in the central region of the third and fourth quadrants. These flavor attributes may be associated with the aroma-active compounds in the third and fourth quadrants. However, the complex relationships among them cannot be further confirmed. This also suggested that relying only on the content of aroma-active compounds and the intensity of aroma attributes, combined with statistical methods, can only tentatively establish the association between aroma-active compounds and aroma attributes and cannot not fully describe the relationship between them. Moreover, aroma-active compounds 17 (3-ethyl-2,5-dimethylpyrazine), 28 (butyric acid), 13 (neo-alloocimene), 12 ((*E*)-2-heptenal), 6 (eucalyptol), 32 (allylacetic acid), 18 (*cis*-linalool oxide), 29 (furfuryl alcohol), 21 (citronellal), 15 (*α*-thujone), 19 (3-buten-1-yl isothiocyanate), 35 (2-acetyl pyrrole), 34 (methyl cyclopentenolone), 30 (piperitone), 27 (terpinen-4-ol), 8 (*γ*-terpinene), 4 (*α*-terpinene), 24 (linalool), 33 (2-hydroxy-3-methyl-2-cyclopentenone), 23 (benzaldehyde), 5 (limonene), and 2 (sabinene) exhibited variable importance for projection (VIP) values greater than 1 (Figure 4b), suggesting a strong association with the flavor of FTOs [23,35]. Of course, the above inference still requires further sensory verification to support it.

### 3.6. Aroma Contribution of Each Compound Determined by OAV

In FFTO and DFTO, there were 18 and 17 compounds with OAV > 1, respectively (Table 2). These aroma-active compounds may significantly contribute to the flavor of FTOs [36]. Among these, the OAVs for *β*-pinene, sabinene, *β*-myrcene, α-terpinene, limonene, eucalyptol, *β*-phellandrene, terpinolene, octanal, *α*-thujone, 3-buten-1-yl isothiocyanate, benzaldehyde, linalool, linalyl acetate, and *β*-caryophyllene in both FFTO and DFTO were all greater than 1. This indicates that these compounds likely make significant contributions to the flavor characteristics of FTO and are important constituents of the *Tengjiao* flavor profile. Specifically, citronellal and terpinen-4-ol exhibited OAVs greater than 1 only in FFTO, while *γ*-terpinene showed OAVs greater than 1 only in DFTO. Therefore, these three aroma compounds may be the factors responsible for the difference in FFTO and DFTO flavors. Higher OAV indicates a greater contribution to the overall flavor profile of the sample. In FFTO, linalool (OAV = 14,063.62), *β*-phellandrene (OAV = 507.11), limonene (OAV = 174.50), sabinene (OAV = 142.91), eucalyptol (OAV = 55.60), 3-phenylpropionitrile (OAV = 26.53), *β*-myrcene (OAV = 21.43), *α*-thujone (OAV = 15.81), *α*-terpinene (OAV = 12.88), and terpinen-4-ol (OAV = 11.15) may play a key role in the overall flavor profile of FFTO. In DFTO, linalool (OAV = 1848.73), *β*-phellandrene (OAV = 369.14), limonene (OAV = 116.11), sabinene (OAV = 77.54), 3-phenylpropionitrile (OAV = 56.80), *α*-terpinene (OAV = 41.80), 3-buten-1-yl isothiocyanate (OAV = 22.35), *β*-myrcene (OAV = 16.62), and *β*-caryophyllene (OAV = 12.73) may play a key role in the overall flavor profile of DFTO. In short, the above aroma-active compounds might theoretically make significant contributions to the flavor of FTOs. However, further sensory experiments are required for support and validation.

### 3.7. Determination and Verification of Key Flavor Compounds by Recombination Experiments and Omission Tests

**Figure 5:**
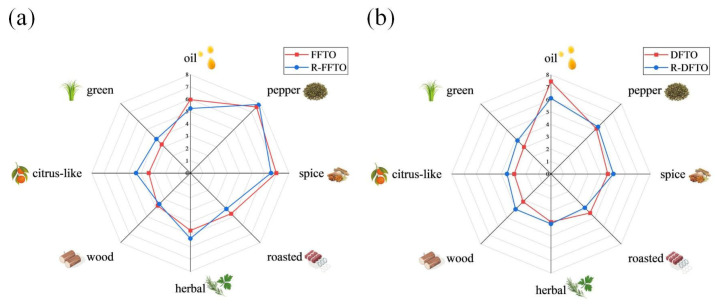
Aroma profiles of FTOs compared to recombinant models. (**a**) FFTO and recombinant model–FFTO (R-FFTO). (A *t*-test was performed: No attributes with *p* < 0.05). (**b**) DFTO and recombinant model–DFTO (R-DFTO). (A *t*-test was performed. oil: *p* < 0.05. FFTO, fresh fried *Tengjiao* oil; DFTO, dried fried *Tengjiao* oil; R-FFTO, recombinant fresh fried *Tengjiao* oil; R-DFTO, recombinant dried fried *Tengjiao* oil).

Currently, recombination experiments and omission tests are widely applied in food flavor research and serve as one of the primary methods for identifying key flavor compounds [37]. Based on the OAV analysis results, we conducted reconstruction experiments using aroma-active compounds with OAV > 1 from both FFTO and DFTO, as well as those for which OAV calculation was not performed due to missing threshold values. The amounts of each aroma-active compound added in the experiment were based on the quantitative results provided in Table 2 via standard curve analysis. The results of the recombinant experiment are shown in Figure 5. In Figure 5a, the recombinant model (R-FFTO) exhibits slightly stronger green, citrus-like, herbal, and pepper notes than FFTO, while its oil, spice, roasted, and wood notes are slightly weaker than FFTO. In Figure 5b, the recombinant model (R-DFTO) exhibits slightly stronger green, citrus-like, wood, herbal, spice, and pepper notes than DFTO, while its oil and roasted notes are slightly weaker than DFTO. The recombinant models are in general agreement with the aroma profile of FTOs, indicating that the recombinations were successful.

Subsequently, we conducted omission tests on R-FFTO and R-DFTO, respectively. Specifically, to further validate the key flavor compounds of FTOs, 22 compounds in recombination experiments were filtered for omission testing, and one of the odorants was omitted from each set of models (M1–M22) (Table 3). A sensory panel of 20 professionally trained panelists was organized to evaluate the difference in aroma between the recombinant model and M1–M22 through a triangle test. This sensory evaluation experiment has also been approved and licensed by the Scientific Research Ethics Committee (Reference number: No. 139 in 2025) mentioned in Section 2.4. As shown in Table 3, in FFTO and DFTO, seven and six compounds showed significance in the triangle test results, respectively. In FFTO, linalool*** (floral, lavender, lemon, sweet), *α*-thujone*** (green, lemon), linalyl acetate*** (sweet, floral), 3-buten-1-yl isothiocyanate* (floral, alcohol), citronellal* (leaf, pepper), *β*-caryophyllene* (spice, bitter, floral), and 3-phenylpropionitrile* (spice) were key flavor compounds that played important roles in flavor formation. In DFTO, *β*-caryophyllene*** (spice, bitter, floral), linalool** (floral, lavender, lemon, sweet), *β*-pinene* (lemon, pine, sweet, bitter), *α*-terpinene* (sweet, lemon), *β*-phellandrene* (mint, sweet) and 3-ethyl-2,5-dimethylpyrazine* (sesame, sweet, oil, roast) were key flavor compounds that played important roles in flavor formation. In particular, linalool and *β*-caryophyllene were significant in both FFTO and DFTO, indicating that they are important flavor compounds within the characteristic flavor profile of *Tengjiao*. The linalool content in DFTO was significantly lower than that in FFTO, indicating that most of the linalool degraded and transformed during the drying process. This further results in a reduction in the characteristic Sichuan pepper flavor in DFTO. Under the influence of thermal oxidation, the C^+^ of linalool could undergo cyclization to form monoterpenes, such as *α*-terpineol, limonene, and terpinolene, and the C^+^ group could further rearrange to form myrcene and *β*-ocimene [17]. *β*-pinene, *α*-terpinene, *β*-phellandrene, *α*-thujone, 3-buten-1-yl isothiocyanate, citronellal, linalyl acetate, 3-phenylpropionitrile, and 3-ethyl-2,5-dimethylpyrazine were the differential key flavor compounds between FFTO and DFTO. *α*-Thujone exhibited extremely high significance only in FFTO, and its concentration was also significantly higher in FFTO than in DFTO; this might be the reason for the slightly stronger green aroma in FFTO. The difference between the OAV analysis results and those from the omission experiment might be attributable to the complex interactions within the food system [38]. In particular, limonene—which has a high OAV among FTOs—was not identified by most participants in the three-point test. It is possible that the combined effects of other compounds have replaced its own effects.

### 3.8. The Enantiomeric Ratio and Its Effect on FTOs’ Flavor

To explore the effect of the enantiomeric ratio in FPO on its flavor profile, we determined the enantiomeric ratios of limonene and linalool, which are abundant in FPO (Table 4). The *t*-test results indicate that there is a significant difference in the enantiomers of linalool between FFTO and DFTO. It has been demonstrated that the processing of tea leaves affects the enantiomeric ratio of chiral compounds within them, which in turn influences the flavor profile [40]. In FTOs, the ratio of (S)-(−)-limonene to (R)-(+)-limonene in FFTO and DFTO was similar, with both being close to 7:3. Due to the high proportion of (S)-(−)-limonene, FFTO and DFTO exhibited a herbal and citrus-like aroma, consistent with our AEDA results. Meanwhile, linalool showed a significant difference in enantiomeric ratio. In both FFTO and DFTO, (R)-(−)-linalool was dominant, accounting for 81.68% in FFTO and 90.83% in DFTO. Due to its high proportion of (R)-(−)-linalool, FFTO and DFTO exhibited distinct sweet and floral notes [20] from linalool, consistent with our AEDA results. In sensory evaluation, the citrus-like attributes of FFTO and DFTO were not significantly different, with FFTO exhibiting a slightly higher level than DFTO. This difference may be attributable to other aroma-active compounds present in FTO, such as γ-terpinene. In addition, FFTO had a slightly stronger wood aroma than DFTO, which may be related to the higher proportion of (S)-(+)-linalool in FFTO compared to DFTO. In addition, FFTO had a slightly stronger wood aroma than DFTO, which may be related to the higher proportion of (S)-(+)-linalool in FFTO compared to DFTO. The main difference between the FFTO and DFTO preparation processes lay in the hot-air drying of the *Tengjiao* fruits. During hot-air drying, linalool may undergo a transformation [17], from which we can infer that hot-air drying may promote the conversion of (S)-(+)-linalool to (R)-(−)-linalool, resulting in the difference in enantiomeric ratio.

## 4. Conclusions

In this study, we compared the flavor differences between FFTO and DFTO by molecular sensory science. First, sensory evaluation was used to compare the flavor profiles of the two samples. In terms of overall flavor profile, FFTO had stronger pepper and spice notes, while DFTO exhibited a more marked oily aroma. Recombination experiments and omission tests were conducted based on the AEDA and OAV results. Linalool and *β*-caryophyllene were both key flavor compounds within the characteristic flavor profile of 2 FTOs. *α*-Thujone, 3-buten-1-yl isothiocyanate, citronellal, linalyl acetate, and 3-henylpropionitrile were key flavor compounds in FFTO, and β-pinene, α-terpinene, β-phellandrene, and 3-ethyl-2,5-dimethylpyrazine were key flavor compounds in DFTO. Furthermore, chiral analysis revealed that the predominant enantiomers in FTO were primarily (R)-(−)-linalool and (S)-(−)-limonene. In conclusion, in this experiment, we determined and compared the differences in key flavor compounds between FFTO and DFTO and analyzed the enantiomeric ratios of limonene and linalool. Based on the results, we explained the reasons for using both fresh and dried *Tengjiao* in spicy dishes from a flavor perspective. This study provides theoretical guidance for the production of spicy dishes. In the future, we can perform more in-depth research, such as exploring how the ratio of fresh *Tengjiao* to dried *Tengjiao* affects the flavor of dishes. This study provides preliminary insights into the flavor differences between FFTO and DFTO at the molecular level. However, the exact mechanism has not yet been elucidated. A clear mechanism will provide valuable guidance for quality control in FTO.

## Figures and Tables

**Table 1:** The results of AEDA for FFTO and DFTO.

RT	Compound	CAS	Log_3_FD		Odor Description
			FFTO	DFTO	
6	Hexanal	66-25-1	1	2	green, sweet
6.5	*β*-Pinene	127-91-3	6	-	lemon, pine, sweet, bitter
7.07	Sabinene	3387-41-5	7	6	sweet, bitter, wood
8.95	*β*-Myrcene	123-35-3	7	6	herb, pepper, green
9.31	*α*-Terpinene	99-86-5	4	-	sweet, lemon
10.18	Limonene	5989-27-5	9	7	citrus, herb
10.36	Eucalyptol	470-82-6	6	3	green, mint, pepper
10.49	*β*-Phellandrene	555-10-2	0	3	mint, sweet
11.62	4-Octanone	589-63-9	-	2	sweet, fruit
12.41	*γ*-Terpinene	99-85-4	4	3	pepper, citrus, spice
13.13	Styrene	100-42-5	-	3	sweet
14.26	Terpinolene	586-62-9	-	3	sweet, pine
14.6	Unknown		-	3	roast
14.89	Octanal	124-13-0	7	4	green, sweet
15.5	Unknown		-	5	mold, mushroom
16.61	(*E*)-2-Heptenal	18829-55-5	7	5	fruit, sour, chocolate
19.464	Allo-ocimene	673-84-7	-	4	pepper, stinky
19.5	Neo-alloocimene	7216-56-0	3	-	bitter, pepper, stinky, lemon
20.25	Unknown		-	3	floral, green, wine
20.77	Nonanal	124-19-6	2	3	fat, floral, pepper, nut
21.6	*α*-Thujone	546-80-5	8	-	green, lemon
22.51	(E)-2-Octenal	2548-87-0	4	3	spice, herb, nut
22.75	Thujone	1125-12-8	8	8	cucumber, green
23.22	3-Ethyl-2,5-diMethylpyrazine	13360-65-1	-	6	sesame, sweet, oil, roast
23.33	cis-Linalool Oxide	5989-33-3	3	-	pepper, sweet
24.4	3-Buten-1-yl Isothiocyanate	3386-97-8	-	3	floral, alcohol
24.3	Acetic acid	64-19-7	6	3	vinegar, sour
24.86	trans-Sabinene hydrate	17699-16-0	4	-	milk, sweet
25.72	Citronellal	106-23-0	4	-	leaf, pepper
26.217	Unknown		-	4	moldy
26.31	(*E*,*E*)-2,4-Heptadienal	4313-03-5	-	0	green, almond, nut, oil
27.11	2-Acetylfuran	1192-62-7	5	3	balsamic, pepper
27.72	Benzaldehyde	100-52-7	7	7	bitter, sauce
28.528	Unknown		-	2	sesame, almonds, oil
30.3	Linalool	78-70-6	10	8	floral, lavender, lemon, sweet
30.43	Linalyl acetate	115-95-7	3	-	sweet, floral
31.03	5-Methyl furfural	620-02-0	-	0	almond milk, sweet, spicy
31.41	*β*-Caryophyllene	87-44-5	3	2	spice, bitter, floral
32.5	Terpinen-4-ol	562-74-3	1	4	must, smoke
33.23	Myrtenal	564-94-3	3	-	burnt
33.48	*γ*-Butyrolactone	96-48-0	-	2	peanuts, rice
34.269	Butyric Acid	107-92-6	3	-	cheese, sour, sweet
34.69	trans-2-Decenal	3913-81-3	-	1	sour, pepper, green, cilantro
35.38	α-Caryophyllene	6753-98-6	2	-	pepper, wood
36.5	Furfuryl alcohol	98-00-0	3	3	burnt, rice, bean, yeast powder
37.1	Unknown		3	3	fermented black beans, soy sauce, sour
37.94	*α*-Terpineol	98-55-5	1	0	anise, oil, almonds
38.192	Unknown		3	-	fermentation
38.55	Piperitone	89-81-6	3	-	lemon, pepper, green, paste
40.267	(*E*)-4-Undecenal	68820-35-9	-	1	stinky, salted fish, sour
40.512	Unknown		2	4	rice, bean, stinky
43.19	Unknown		3	4	green, sour
43.61	trans,trans-2,4-Decadienal	25152-84-5	3	9	fat, oil, pepper
43.943	Allylacetic acid	591-80-0	-	8	fruit, sweet
44.81	2-Hydroxy-3-Methyl-2-cyclopentenone	765-70-8	-	3	wood, bitter, floral
44.84	Methyl cyclopentenolone	80-71-7	3	-	pepper, green lemon
45.383	Unknown		3	8	dust, wheat
46.022	Unknown		3	0	sweet
46.47	Unknown		-	5	bitter, burnt
51.96	2-Acetyl pyrrole	1072-83-9	-	8	roast
54.53	3-Phenylpropionitrile	645-59-0	3	3	spice
54.64	Unknown		-	6	sweet, wheat
55.31	cis-Nerolidol	142-50-7	1	-	spice, floral
58.38	Unknown		3	8	Perilla, plastic
63.68	Indole	120-72-9	1	-	mothball

“-” indicates that the compound was not detected. The experiment was conducted once by each of the three participants. (FFTO, fresh fried *Tengjiao* oil; DFTO, dried fried *Tengjiao* oil; AEDA, aroma extract concentration analysis; RT, retention time; FD, flavor dilution).

**Table 3:** The significance of each compound in the omission tests.

No.	Compound	Number of Correct Answers		Significance	
		FFTO	DFTO	FFTO	DFTO
M1	*β*-Pinene	8/20	11/20	NS	*
M2	Sabinene	6/20	8/20	NS	NS
M3	*β*-Myrcene	8/20	6/20	NS	NS
M4	*α*-Terpinene	4/20	11/20	NS	*
M5	Limonene	4/20	9/20	NS	NS
M6	Eucalyptol	3/20	6/20	NS	NS
M7	*β*-Phellandrene	9/20	11/20	NS	*
M8	Terpinolene	4/20	6/20	NS	NS
M9	Octanal	5/20	6/20	NS	NS
M10	Neo-alloocimene	7/20	-	NS	-
M11	*α*-Thujone	15/20	8/20	***	NS
M12	3-Buten-1-yl Isothiocyanate	12/20	6/20	*	NS
M13	Citronellal	11/20	-	*	-
M14	Benzaldehyde	8/20	2/20	NS	NS
M15	Linalool	16/20	13/20	***	**
M16	Linalyl acetate	14/20	5/20	***	NS
M17	*β*-Caryophyllene	11/20	14/20	*	***
M18	Terpinen-4-ol	6/20	-	NS	-
M19	3-Phenylpropionitrile	11/20	4/20	*	NS
M20	*γ*-Terpinene	-	5/20	-	NS
M21	3-Ethyl-2,5-diMethylpyrazine	-	11/20	-	*
M22	Allylacetic acid	-	4/20	-	NS

“-” indicates that the compound was not detected or the odor threshold was not found. The experiment is based on the sensory analysis method—the triangle test (GB/T 12311-2012 [39]). The experiment was conducted once by each of the 20 participants. *** 0.1% significance level. ** 1% significance level. * 5% significance level. NS, no significant difference. (FFTO, fresh fried *Tengjiao* oil; DFTO, dried fried *Tengjiao* oil).

**Table 4:** The enantiomeric ratio of limonene and linalool in FFTO and DFTO.

Compound	CAS	Odor Description [15]	Enantiomeric Ratio (%)	
			FFTO (%)	DFTO (%)
(S)-(−)-Limonene	5989-54-8	lemon-like, piney	70.11 ± 2.61	70.96 ± 0.82
(R)-(+)-Limonene	5989-27-5	citrus-like	29.89 ± 2.61	29.98 ± 0.82
(S)-(+)-linalool *	126-90-9	woody, lavender-like	18.32 ± 2.34	9.17 ± 1.2
(R)-(−)-linalool *	126-91-0	sweet, floral, petitgrain-like	81.68 ± 2.34	90.83 ± 1.2

“*” indicates that *p* < 0.05 in the *t*-test, indicating a statistically significant difference. The contents are expressed as the average of three experiments, presented as the mean ± standard deviation. (FFTO, fresh fried *Tengjiao* oil; DFTO, dried fried *Tengjiao* oil).

## Data Availability

The original contributions presented in this study are included in the article. Further inquiries can be directed to the corresponding authors.

## Abbreviations

The following abbreviations are used in this manuscript:

ZA	*Zanthoxylum armatum* DC.
FTO	Fried *Tengjiao* oil
FFTO	Fresh fried *Tengjiao* oil
DFTO	Dried fried *Tengjiao* oil
ZB	*Zanthoxylum bungeanum* Maxim.
OAV	Odor activity value
ZS	*Zanthoxylum schinifolium* sieb. et Zucc.
VOCs	Volatile organic compounds
SAFE	Solvent-assisted flavor evaporation
GC-MS	Gas chromatography–mass spectrometry
QDA	Quantitative descriptive analysis
AEDA	Aroma extract concentration analysis
FD	Flavor dilution
PLSR	Partial least squares regression
VIP	Variable importance for the projection

## Appendix A

**Table A1:** The results of GC-MS for FFTO and DFTO.

RT	Compound	CAS	FFTO	DFTO	Identification
3.66	Pentanal	110-62-3		√	MS, RI, S
4.23	tert-Amyl alcohol	75-85-4	√	√	MS, RI, S
4.32	*α*-Pinene	80-56-8	√	√	MS, RI, S
4.46	*α*-Phellandrene	99-83-2	√	√	MS, S
4.83	1-Propanol	71-23-8	√		MS, RI, S
5.31	Camphene	79-92-5	√		MS, RI, S
6	Hexanal	66-25-1	√	√	MS, RI, S, O
6.5	*β*-Pinene	127-91-3	√	√	MS, RI, S, O
7.07	Sabinene	3387-41-5	√	√	MS, RI, S, O
7.4	Ethylbenzene	100-41-4		√	MS, RI, S
7.55	trans-2-Pentenal	1576-87-0		√	MS, RI, S
7.62	*p*-Xylene	106-42-3	√	√	MS, RI, S
8.29	1-Butanol	71-36-3	√		MS, RI, S
8.88	1-Penten-3-ol	616-25-1		√	MS, RI, S
8.95	*β*-Myrcene	123-35-3	√	√	MS, RI, S, O
9.31	*α*-Terpinene	99-86-5	√	√	MS, RI, S, O
9.58	*o*-Xylene	95-47-6	√	√	MS, RI, S
9.74	3-Butenenitrile	109-75-1		√	MS, RI, S
9.83	Heptanal	111-71-7		√	MS, RI, S
10.18	Limonene	5989-27-5	√	√	MS, RI, S, O
10.36	Eucalyptol	470-82-6	√	√	MS, RI, S, O
10.49	*β*-Phellandrene	555-10-2	√	√	MS, RI, S, O
11.21	(*E*)-2-Hexenal	6728-26-3		√	MS, RI, S
11.62	4-Octanone	589-63-9	√	√	MS, RI, S, O
12.02	2-pentyl-Furan	3777-69-3		√	MS, RI, S
12.23	*trans*-*β*-Ocimene	3779-61-1	√		MS, RI, S
12.41	*γ*-Terpinene	99-85-4	√	√	MS, RI, S, O
13.13	Styrene	100-42-5	√	√	MS, RI, S, O
13.4	2-Methylpyrazine	109-08-0		√	MS, RI, S
13.67	*p*-Cymene	99-87-6	√	√	MS, RI, S
14.12	3-Methylcrotononitrile	4786-24-7	√	√	MS
14.26	Terpinolene	586-62-9	√	√	MS, RI, S, O
14.89	Octanal	124-13-0	√	√	MS, RI, S, O
16.25	2,5-Dimethyl pyrazine	123-32-0		√	MS, RI, S
16.61	(*E*)-2-Heptenal	18829-55-5	√	√	MS, RI, S, O
17.54	6-Methyl-5-hepten-2-one	110-93-0		√	MS, RI, S
18	5-Hexenenitrile	5048-19-1	√	√	MS, RI, S
19.464	2,6-DiMethyl-2,4,6-octatriene	673-84-7		√	MS, O
19.5	Neo-alloocimene	7216-56-0	√		MS, S, O
20.77	Nonanal	124-19-6	√	√	MS, RI, S, O
21.6	*α*-Thujone	546-80-5	√	√	MS, RI, S, O
22.51	(*E*)-2-Octenal	2548-87-0	√	√	MS, RI, S, O
22.75	Thujone	1125-12-8	√	√	MS, RI, S, O
23.22	3-Ethyl-2,5-diMethylpyrazine	13360-65-1		√	MS, RI, S, O
23.33	*cis*-Linalool Oxide	5989-33-3	√		MS, RI, S, O
24.3	Acetic acid	64-19-7	√	√	MS, S, O
24.4	3-Buten-1-yl Isothiocyanate	3386-97-8	√	√	MS, RI, S, O
24.86	*trans*-Sabinene hydrate	17699-16-0	√		MS, O
25	Furfural	98-01-1	√	√	MS, RI, S
25.72	Citronellal	106-23-0	√		MS, RI, S, O
26.31	(*E*,*E*)-2,4-Heptadienal	4313-03-5	√	√	MS, RI, S, O
27.11	2-Acetylfuran	1192-62-7	√	√	MS, RI, S, O
27.72	Benzaldehyde	100-52-7	√	√	MS, RI, S, O
30.3	Linalool	78-70-6	√	√	MS, RI, S, O
30.43	Linalyl acetate	115-95-7	√	√	MS, RI, S, O
31.03	5-Methyl furfural	620-02-0	√	√	MS, RI, S, O
31.41	*β*-Caryophyllene	87-44-5	√	√	MS, RI, S, O
32.5	Terpinen-4-ol	562-74-3	√	√	MS, RI, S, O
33.23	Myrtenal	564-94-3	√		MS, RI, O
33.48	Gamma Butyrolactone	96-48-0		√	MS, S, O
34.269	Butyric Acid	107-92-6	√		MS, S, O
34.69	*trans*-2-Decenal	3913-81-3		√	MS, RI, S, O
35.38	*α*-Caryophyllene	6753-98-6	√	√	MS, RI, S, O
35.41	N-Methyl-2-pyrrolidone	872-50-4		√	MS, RI, S
36.5	Furfuryl alcohol	98-00-0	√	√	MS, RI, S, O
37.62	Germacrene D	23986-74-5	√	√	MS, RI, S
37.94	*α*-Terpineol	98-55-5	√	√	MS, RI, S, O
38.55	Piperitone	89-81-6	√		MS, RI, S, O
40.267	(*E*)-4-Undecenal	68820-35-9		√	MS, O
40.38	2(5H)-Furanone	497-23-4		√	MS, RI, S
41.24	Methyl phenylacetate	101-41-7	√		MS, RI, S
42.9	Myrtenol	515-00-4	√		MS, RI, S
43.61	*trans*,*trans*-2,4-Decadienal	25152-84-5	√	√	MS, RI, S, O
43.943	Allylacetic acid	591-80-0		√	MS, S, O
44.1	Phenethyl acetate	103-45-7	√		MS, RI, S
44.81	2-Hydroxy-3-Methyl-2-cyclopentenone	765-70-8		√	MS, RI, S, O
44.84	Methyl cyclopentenolone	80-71-7	√		MS, RI, S, O
49.04	Phenylethyl Alcohol	60-12-8	√		MS, RI, S
51.96	2-Acetyl pyrrole	1072-83-9		√	MS, RI, S, O
54.53	3-Phenylpropionitrile	645-59-0	√	√	MS, RI, S, O
55.31	cis-Nerolidol	142-50-7	√		MS, O
63.68	Indole	120-72-9	√		MS, RI, S, O

“√” indicates that the compound was detected.
